# Examining the Relationship between Death Anxiety and Well-Being of Frontline Medical Staff during the COVID-19 Pandemic

**DOI:** 10.3390/ijerph192013430

**Published:** 2022-10-18

**Authors:** Na Zhao, Beikun Liu, Yiheng Wang

**Affiliations:** Department of Psychology, School of Sociology and Psychology, Central University of Finance and Economics, Beijing 100081, China

**Keywords:** death anxiety, hedonic well-being, eudaimonic well-being, narcissistic personality, medical staff in COVID-19

## Abstract

To examine the well-being of medical staff during the COVID-19 pandemic, we conducted a survey of 705 medical staff who were involved in anti-epidemic work in China from 20 February to 16 March 2020. The findings of the present study showed a “psychological typhoon eye” effect in which the medical staff in areas with a high contagion rate showed a significantly lower level of death anxiety than those in low-contagion regions. We also found a significant negative relationship between death anxiety and hedonic well-being, but there was no relationship between death anxiety and eudaimonic well-being. Moreover, the results revealed that a narcissistic personality moderates the relationships between death anxiety and the two types of well-being. For those who had higher narcissistic personality scores, death anxiety had no negative effect on their well-being. The findings of the present study can help us to better understand the life profiles of medical staff and can also provide some practical implications for understanding the life conditions of medical staff when facing a great health crisis.

## 1. Introduction

The World Health Organization (WHO) declared the COVID-19 outbreak, which was first detected in Wuhan and rapidly spread to the rest of the world, a global health emergency in January 2020 and a global pandemic in March 2020. It has impacted millions worldwide, invoking feelings of death anxiety, uncertainty and fear, depression, insomnia, and other psychological and behavioral problems [1,2]. People were overwhelmed by the possibility of death for others and themselves, which evoked death anxiety [3]. The well-being profiles of the medical staff who worked on the frontline during the COVID-19 pandemic should be given more attention, as they risked their lives fighting the highly contagious novel disease. They frequently encountered death when working directly with patients at the emergent stage of the pandemic. The purpose of the present study was to examine (1) the hedonic and eudaimonic well-being of the medical staff who worked on the frontline throughout the COVID-19 pandemic; (2) the relationships between death anxiety and the two types of well-being; and (3) the possible moderators of the relationships between death anxiety and the two types of well-being.

### 1.1. Hedonic and Eudaimonic Well-Being

Well-being is a complex construct that refers to optimal psychological experiences and functioning [4]. Great strides in well-being research have been made in the field of positive psychology, and researchers have posited theories of hedonic well-being and eudaimonic well-being.

Hedonic well-being focuses on subjective cognitive-affective experiences, whereas eudaimonic well-being reflects the “true self”, including experiences of meaning and purpose in life [5]. Hedonic well-being consists of subjective happiness and experiences of pleasure versus displeasure when considering one’s life [6], which can be seen as subjective well-being [7]. Life satisfaction, as one of the key elements of subjective well-being, has been conceptualized and measured through happiness by many researchers [8]. In contrast to hedonic well-being, eudaimonic well-being focused on life meaning, authenticity and purposefulness [9]. Meaning was widely considered as the proxy for all things eudaimonic; it served as a good factor for eudaimonic well-being [10]. Eudaimonic well-being is achieved when people’s life activities are congruent with their deeply held values. It is believed to lead to success in the face of existential challenges in life [11]. Ryff and Keyes [12] stated that life purpose and personal growth were two important functions of eudaimonic well-being. Previous studies showed that many people who were forced to deal with trauma ultimately experienced growth and found meaning in their suffering [13].

The COVID-19 pandemic has seriously influenced people’s well-being [14,15]. During the early stage of the pandemic, when patients were rushed to the hospital in larger numbers than normal and little was known about the disease, a significant strain was placed on medical staff. However, few studies have focused on the well-being of medical staff [16], even though frontline health care professionals working with COVID-19 patients have shown higher levels of stress, burnout, secondary trauma, anxiety, and depression [17]. Most studies have focused on hedonic well-being, and even fewer have studied frontline health workers, who risk their health and lives for the sake of others.

### 1.2. Death Anxiety and Well-Being among Medical Staff during the COVID-19 Pandemic

According to terror management theory [18], individuals have an inherent fear of inevitable death. In order to manage this potential terror, people need to achieve happiness and meaning in life. Studies showed that heightened awareness of death impeded well-being [19]. Death anxiety refers to a negative state of mind regarding death and dying, derived from a perceived threat to one’s existence [20]. It can be heightened following traumatic life events such as disasters [21] and is often associated with psychiatric disorders such as PTSD [22]. Juhl and Routledge [23] concluded that experimentally heightening death awareness undermined participants’ life satisfaction. Other studies have also demonstrated that individuals who experience more anxiety when their mortality is emphasized have lower psychological well-being [23,24,25]. Following the outbreak of COVID-19, reminders about death were all around. The pandemic caused dramatic collapses in health care systems and led to significant loss of health and life [25]. The reminder effects were even more profound for the medical staff who worked on the frontline because they came into frequent contact with dying patients. In this study, we argue that death anxiety during the COVID-19 pandemic decreased the hedonic well-being of medical staff.

However, the relationship between death anxiety and eudaimonic well-being may differ from the relationship between death anxiety and hedonic well-being. First, the high-risk situation of the COVID-19 pandemic could not only produce psychological stress but could also provide an opportunity for posttraumatic growth [26]. Such situations may provoke reexamination and reappraisal of an individual’s life and promote their recognition of growth in domains such as personal strength, relationships with others, and appreciation of life [27]. A previous study showed that many individuals who struggled to deal with trauma ultimately found meaning in their suffering [28]. Second, meaning in life may buffer death-related anxiety and uncertainty when people’s mortality is highlighted. Yaakobi [29] examined the relationship between the fear of death and work. The study found that salient mortality led to the participants having a higher desire to work (Study 1), and activating thoughts of fulfilling the desire to work reduced the accessibility of death-related thoughts (Study 2). The results indicated that valuable work, such as work during the COVID-19 pandemic, serves as a buffer mechanism for death anxiety. Landau et al. [30] stated that people protect themselves from the awareness of personal mortality by perceiving their actions as purposeful and connected to their broad, temporally extended self-concept [30,31]. Third, a few studies have also indicated there is a positive link between death and meaning in life from the perspective of scarcity. Death, essentially the termination of life, represents the scarcity of life [32], and limitation of life could increase the thinking about life, prompting individuals to enhance their potentials and to find meaning in life [33].

We proposed that medical staff who worked on the frontline during the COVID-19 pandemic may resemble Baumeister’s [34] depiction of a lower hedonic but relatively high eudaimonic life among those who sacrificed their personal pleasures for the good of society.

**Hypothesis** **1.** *Death anxiety evoked by the pandemic would have a main effect on hedonic well-being, but have no main effect on eudaimonic well-being*.

### 1.3. The Role of the Narcissistic Personality

Studies on the relationship between personality traits and well-being have mainly focused on general factors, such as the Big Five personality traits [35,36,37]. Few studies have investigated the dark side of the human personality, especially in the context of threats. Narcissism is a dark personality trait characterized by a lack of empathy, a grandiose self-view, and egotism [38].

Although regarded as a “dark trait”, studies have shown that narcissism has some adaptive functions, especially in the context of difficult circumstances. For example, narcissism may facilitate the active and passive accrual of social networks, which may buffer deleterious health outcomes [39,40]. Indeed, narcissism is correlated with subjective well-being [41]. Jonason et al. [42] argued that narcissism is related to positive health outcomes and a greater life expectancy, given the value that individuals who are high in narcissism place on social connections (in Study 3). They also argued that narcissism is related to a slow life history strategy, which makes it possible that narcissism is linked to enhanced life expectancy despite the reasons they may desire the presence of others in their lives. Being mentally tough may protect people from feeling vulnerable when faced with the possibility of death. Narcissism indicates mental toughness, which, in turn, reduces perceived stress [43] and increases psychological well-being [44].

One recent study showed that narcissists were more likely to experience eudaimonic well-being, as they were more likely to adopt a long-term view of life [45]. Accordingly, narcissists also tend to rely on overt strategies to regulate the self and appear more powerful (e.g., self-enhancement) [46]. We argue that a narcissistic personality may protect people from feeling vulnerable when faced with the possibility of death. Specifically, the negative predictive effect of death anxiety on life satisfaction was lower or did not exist in individuals who had a high narcissistic personality score.

**Hypothesis** **2.** *Narcissism plays a moderating role between death anxiety and the two types of well-being. Specifically, death anxiety has a significant negative effect on the two kinds of well-being for people with a lower narcissistic personality score compared to those with a higher narcissistic personality score*.

## 2. Materials and Methods

### 2.1. Data Collection and Sample

The survey period of this study was from 20 February to 16 March 2020, when quarantine was strictly regulated throughout China. This period was marked by peak COVID-19 infection and mortality rates in Wuhan, where severe cases accounted for 20~30% of the country [47], let alone suspected cases and confirmed cases, and the whole society was highly alarmed. Since Wuhan was the center of the pandemic, experiencing higher risk and stress [48], its citizens may have shown psychological expressions different from those of other areas with lower risk. Therefore, we proposed that the location indicator can be an important covariate. We surveyed a sample of medical staff who worked on the frontline in 21 provinces, which included Wuhan (the high-risk province that was most severely affected by COVID-19 at the time) and other relatively low-risk provinces, such as Beijing, Guangdong, Jiangsu, Chongqing, Zhejiang, and Hainan. During the pandemic, it was hard for us to directly contact the medical staff who were facing such a serious situation on the frontline. Thus, the participants were recruited through purposive and snowball sampling [49], drawing on the authors’ connections with the frontline medical staff. Upon providing informed consent to participate in the study, each potential participant received a link to the online survey. Respondents were given a gift coupon (approximately $3) for completing the questionnaires. The study was supported by the Emergency Project to Provide Psychological Assistance by the Central University of Financial Economics.

### 2.2. Measurement

The Cronbach’s alpha value of the scales in the present study all reached 0.80, which meant the scales were of good reliability and high internal consistency [50].

#### 2.2.1. Death Anxiety

To measure death anxiety, we used a Death Anxiety Survey Schedule comprising a set of 10 questions that had been validated in Chinese [51]. A sample question included “Do you think that the word ’death’ is a cause of worry for you?” On this scale, an answer of “yes” was counted as 1 point for each question. Question 8 (“Do you feel normal after the death of your loved ones?”) was reverse scored, and Question 3 (“Do you want to come again in the world?”) was neutral and not included in the total score. Higher scores indicated higher death anxiety. Cronbach’s alpha for the scale used in the present study was 0.88.

#### 2.2.2. Hedonic Well-Being

Life satisfaction, a significant predictor of hedonic well-being, was the most common assessment of hedonic well-being [4,52]. Therefore, we adopted a validated Chinese version of the Satisfaction With Life Scale (SWLS) comprising five items to measure hedonic well-being. We asked the participants to indicate the extent to which they agreed or disagreed with the items on this scale (e.g., “I am satisfied with my life”) on a 7-point Likert scale (1 = “completely disagree”; 7 = “completely agree”), where higher scores indicated greater hedonic well-being. Cronbach’s alpha for this scale in the present study was 0.84.

#### 2.2.3. Eudaimonic Well-Being

Meaning in life linked eudaimonia, and it also empirically fitted the definition of eudaimonia [53]. The participants completed the validated Chinese version of the 10-item Meaning in Life Questionnaire (MLQ), which represented eudaimonic well-being [54]. MLQ contains two dimensions, which are presence of meaning and search for meaning. Presence of meaning is defined as “the degree to which people perceive their lives as comprehensible and significant” [55]. Search for meaning is defined as the “strength, intensity, and activity of people’s desire and efforts to establish and achieve goals and missions” [56]. The sample items were “I understand my life’s meaning” and “I am always searching for something that makes my life feel significant”. We asked the participants to indicate the extent to which they agreed or disagreed with each statement on a 7-point scale (1 = “completely disagree”; 7 = “completely agree”), where higher scores indicated greater eudaimonic well-being. Cronbach’s alpha for this scale in the present study was 0.88.

#### 2.2.4. Narcissistic Personality

Narcissistic personality scores were measured by a widely used narcissistic subscale of *the Dirty Dozen*, consisting of four items measuring grandiose narcissism [57], which has been verified to be reliable over time and across a number of tests. Participants were asked to indicate the extent to which they agreed or disagreed with the items (e.g., “I want people to pay attention to me”) on a 7-point Likert scale (1 = “completely disagree”; 7 = “completely agree”), where higher scores indicated a higher level of narcissism. Cronbach’s alpha for this scale in the present study was 0.86.

### 2.3. Statistical Analysis

The data were analyzed on a personal computer using the SPSS statistical software package (version 20.0 for Windows created by Nie, Hull and Bent from IBM Corp., Armonk, NY, USA). We first conducted a correlation analysis and T-test to preliminarily examine the relationships among the variables. The main and interaction effects were then explored using process 3.2 package created by Hayes from Unity Technologies, San Francisco, CA, USA. We reported the estimated values, effect sizes and confidence intervals for the different effects. There was a significant effect if the confidence interval excluded 0, with the *p* value less than 0.05.

## 3. Results

### 3.1. Characteristics of the Sample

We used G* power 3.1.9.7 created by the University of Duesseldorf, Düsseldorf, Germany to estimate sample size, in which we set α as 0.05 and the power as 0.80. The power analysis revealed that 701 participants could yield an effect size of *f*^2^ = 0.45.

The sample of the present study consisted of 705 medical staff (both doctors and nurses, 223 females, *M_age_* = 35.27 years, *SD* = 7.98), of whom 359 were from hospitals in Wuhan (the epidemic center of the pandemic, a high-risk area), and the remaining 349 were from other areas (low-risk areas).

### 3.2. The Life Profiles of Medical Staff during the COVID-19 Pandemic

The correlations between variables are shown below (Table 1). We tested the assumptions, and the results showed that the absolute value of “Skewness” < 2 and “Kurtosis” < 7, which meant the data met normal distribution [58]. There were no age effects on death anxiety, hedonic well-being or eudaimonic well-being (*p* > 0.05). The results of the T test showed that there were no significant differences between sexes in either hedonic well-being, *t*(703) = 0.77, *p* = 0.44, or eudaimonic well-being, *t*(703) = 0.62, *p* = 0.54. Females had lower narcissistic personality scores than males, *t*(703) = 2.29, *p* < 0.05.

The results showed that the overall hedonic well-being score was 4.11 (*SD* = 1.48) and the overall eudaimonic well-being score was 4.97 (*SD* = 1.01) on a 1–7-point scale. There was a significant negative correlation between death anxiety and hedonic well-being (*r* = −0.15, *p* < 0.01), but there was no correlation between death anxiety and eudaimonic well-being (*r* = 0.06, *p* = 0.88). To the two subscales of eudaimonic well-being, the results demonstrated that there was a significant negative association between death anxiety and presence of meaning (*r* = −0.11, *p* < 0.05), but had no significant association with search for meaning (*r* = −0.07, *p* > 0.05). Furthermore, the results showed a significant positive correlation between the two types of well-being (*r* = 0.51, *p* < 0.01).

**Figure 1:**
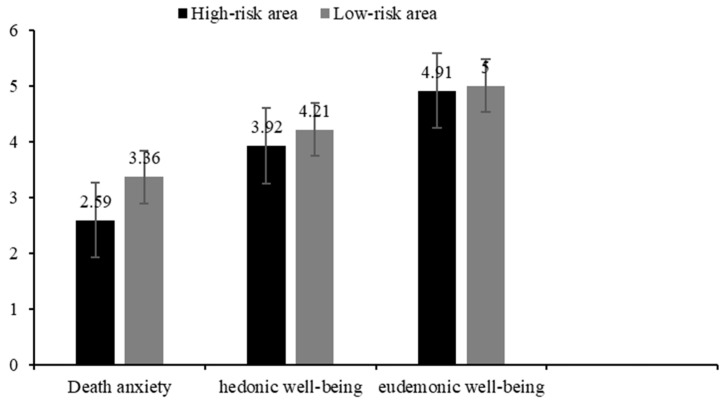
Descriptive Results of Different Variables in Different Locations.

We explored whether there were significant differences between the high-risk area and the low-risk areas. We recoded the epidemic center, Wuhan, as “1” and the other surrounding areas as “0”. The results revealed a significant location effect on death anxiety and hedonic well-being. The results showed that the medical staff who worked in Wuhan (the epidemic center) experienced lower death anxiety (*M* = 2.59, *SD* = 1.91) than those who worked in other areas (*M* = 3.36, *SD* = 2.09), *t*(703) = −5.08, *p* < 0.01, *d* = −0.38, 1 − *β* = 0.97. Similarly, they also had significantly lower hedonic well-being (*M* = 3.92, *SD* = 1.49) than medical staff who worked in other areas (*M* = 4.21, *SD* = 1.58), *t*(703) = 2.02, *p* < 0.01, *d* = −0.19, 1 − *β* = 0.81 (see Figure 1). However, there was no such different-area effect on eudaimonic well-being. Although the medical staff who worked in Wuhan experienced lower eudaimonic well-being (*M* = 4.91, *SD* = 0.95) than those who worked in other areas (*M* = 5.00, *SD* = 1.06), the difference was not significant, *t*(703) = 1.61, *p* = 0.11. However, further analysis of the two subscales found no differences between medical staff who worked in Wuhan (*M* = 4.89, *SD* = 1.16) and those who worked in other areas (*M* = 4.72, *SD* = 1.19) in the search for meaning *t*(703) = 1.05, *p* = 0.29, but a difference in the presence of meaning.

### 3.3. The Relationship between Death Anxiety, Narcissistic Personality, and Well-Being

We used the process 3.2 package, chose Model 1 and adopted 5000 bootstrap sampling to explore a regression model that included main effect and interactive effect of death anxiety and narcissism on the two types of well-being. After statistically adjusting for the effect of location, the results showed that there was a significant main effect of death anxiety on hedonic well-being, *β* = −0.26, *t* = −3.41, *p* < 0.01, 95% CI [−0.42, −0.13]. However, the main effect of death anxiety on eudaimonic well-being was nonsignificant, *β* = −0.11, *t* = −1.92, *p* = 0.11, 95% CI [−0.29, 0.09]. Additionally, there was a main effect of narcissism on hedonic well-being, *β* = −0.14, *t* = −2.25, *p* < 0.01, 95% CI [−0.26, −0.02], but there was no main effect on eudaimonic well-being, *β* = −0.09, *t* = −1.36, *p* = 0.27, 95% CI [−1.11, 0.45]. That is to say, death anxiety during the pandemic only reduced individuals’ hedonic well-being; narcissism only improved individuals’ hedonic well-being.

**Figure 2:**
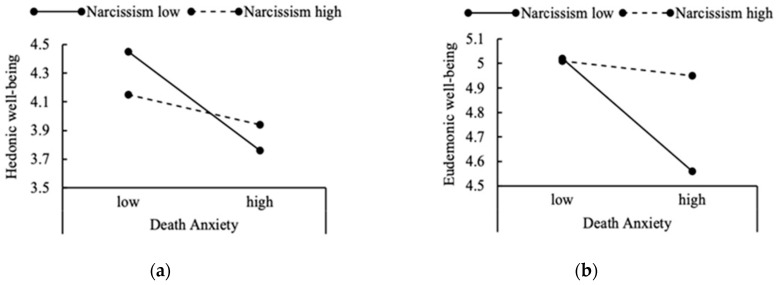
The Simple Slope Analysis, for Individuals ±1 *SD* From the Mean of Well-being on Narcissism: (**a**) the Interaction Effect on Death Anxiety and Hedonic Well-being. (**b**) the Interaction Effect on Death Anxiety and Eudaimonic Well-being.

The results also showed that having a narcissistic personality, to some extent, moderated the association between death anxiety and hedonic well-being, *β* = 0.06, *t* = 2.84, *p* < 0.01, 95% CI [0.01, 0.09], 1 − *β* = 0.99 (See Table 2). The results of a simple slope test showed that for those who had a lower narcissistic personality score (the solid line), death anxiety was a negative predictor of hedonic well-being (*β* = −0.17, *t* = −4.06, *p* < 0.01, 95% CI [−0.25, −0.09]). Conversely, for those who had a higher narcissistic personality score (the dotted line), the results showed that there was no effect of death anxiety on hedonic well-being (*β* = −0.05, *t* = −1.45, *p* = 0.26, 95% CI [−0.12, 0.02]) (See Figure 2a).

We also found a slight interaction effect of death anxiety and narcissism on eudaimonic well-being, *β* = 0.03, *t* = 1.99, *p* < 0.05, 95% CI [0.01, 0.05]. For those who had a lower narcissistic personality score (the real line), death anxiety also had a slightly significant effect on eudaimonic well-being, *β* = −0.11, *t* = −4.09, *p* < 0.01, 95% CI [−0.17, −0.06]. Conversely, for those who had a higher narcissistic personality score (the dotted line), the results showed that there was no effect of death anxiety on eudaimonic well-being, *β* = −0.01, *t* = −0.64, *p* = 0.49, 95% CI [−0.12, 0.02] (See Figure 2b).

## 4. Discussion

We surveyed 705 frontline medical staff from 20 February to 16 March 2020, a period marked by high incidence of the novel diseases and uncertainty. It was a critical time for treating patients and controlling the spread of the pandemic in China. In addition to being exposed to high-risk conditions for extended periods and even risking their lives to fight the pandemic, many health care professionals found themselves overloaded with stress and separated from their families and support systems while facing these hardships.

The findings of the present study draw a general picture of the well-being of medical staff throughout China during the early stages of the pandemic. First, the results of the present study provide strong evidence for the “psychological typhoon eye” effect on death anxiety among medical staff during the pandemic. The “psychological typhoon eye” showed that the closer the residents were to the center of the devastated area, the less they were concerned about the risk [59,60]. A similar effect was found among residents in Wuhan (the early epidemic center) compared to residents in the rest of China during the COVID-19 pandemic [61]. In the present study, this effect was evidenced among medical staff: medical workers in Wuhan (a high-risk area) reported lower death anxiety than medical workers in the rest of China. We argue that the reason may be that Wuhan is very special in that it was the epicenter of the pandemic and the data were collected from 20 February to 16 March 2020, a period marked by high morbidity of the novel disease, as well as uncertainty. Previous study suggested there were no real differences between health care workers and the general population on death anxiety [62]. However, we did not compare death anxiety between medical staff who worked in high-risk areas, low-risk areas and the general residents at that special time, so we cannot give a more general conclusion. Moreover, compared with their counterparts working in other regions, medical staff in Wuhan had lower life satisfaction, but similar levels of meaning in life. One of the major explanations for this effect is that medical staff became desensitized by repeated exposure and could better prepare for death anxiety [59]. Another reason is the gap between imagining and experiencing: healthcare workers in the epidemic center had a more accurate estimate of the risks based on their actual experiences and more practical knowledge related to COVID-19 infection [63]. These results can help us better understand the profiles of medical staff and suggest different coping strategies that should be used in such a large health crisis.

Second, previous research has shown that women experience higher levels of death anxiety than men [64]. However, the present study did not find a sex difference in self-reported death anxiety. This result is consistent with the findings on death anxiety among the medical student population, which showed no sex difference and remained stable across 6 years [65]. This result indicates that during the COVID-19 pandemic, female doctors and nurses were no more threatened with death and dying than male doctors and nurses.

Third, the results of the present study showed lower life satisfaction but higher meaning in life among medical workers. It comes as no surprise that doctors and nurses working on the front lines of the current pandemic face heightened health threats, experience high levels of death anxiety and stress, and have weakened well-being. However, a less common perspective reveals that the hardship has made their lives much more meaningful. Some studies show that meaning-making is widely considered essential for adjusting to stressful events [66]. Higher meaning in life is related to lower levels of anxiety and distress associated with the COVID-19 pandemic [67], and meaning-making could be used as a vital tool to help society cope with collective threats and challenges [68]. When an individual’s meaning systems have been disrupted by traumatic events, other negative effects may help reinstate it [54]. Although the process of searching for some experiences is unpleasant, the search for value can ultimately lead to an enriched sense of meaning in life. However, for eudaimonic well-being, our study indicates some differences between the presence of meaning and the search for meaning on death anxiety. Prior studies focusing on young adults showed that death anxiety was only negatively associated with search for meaning [69]. However, death anxiety was negatively correlated with both presence of meaning and search for meaning in older adults [70]. Differently from those outcomes, we found that death anxiety was only negatively correlated with the presence of meaning. We considered that as participants in the present study, medical staff are groups who deal with death all year round [71], whose goal and mission are saving life. Although in the pandemic period, close contact with infected patients increased the contagion probability to medical staff, resulting in higher death anxiety [72], their goals were still to save life and to achieve meaning in life [73]. COVID-19 is such an uncertain health emergency that it has caused a surge in the loss of life. Faced with high levels of death anxiety, medical staff were experiencing scarcity of life and psychological distress, thus their presence of meaning could be undermined [74].

Fourth, during the COVID-19 pandemic, the two types of well-being showed different patterns among medical staff. People may accept negative emotions in order to help others, as Florence Nightingale once did and medical staff do today. They sacrifice their personal pleasure to participate constructively in society and make substantial contributions. Tongeren et al. [75] provided indirect evidence that prosociality could enhance the level of eudaimonic well-being. Unlike a hedonic life, which is primarily linked to satisfying one’s own needs, a eudaimonic life involves contributing to the welfare of others or other culturally valued activities. Difficulties within a particular context, such as a pandemic, encourage negative emotions in the moment and decrease hedonic well-being. However, when people face difficulties, they may spend much more time thinking about the past and future from a future perspective, which could encourage them to reestablish eudaimonic well-being [76].

Finally, the findings of the current study also provide a new perspective on narcissism. There is a long line of research linking this personality type to various health outcomes. Previous studies have shown that people who possess any one trait of this personality type seem to have an extremely low regard for other people and score high on disagreeableness, irresponsibility, and low-level empathy [77]. However, from an evolutionary perspective, we provided evidence that a narcissistic personality plays a positive role during a pandemic when people are faced with high levels of threat, such as death anxiety. The negative predictive effect of death anxiety on life satisfaction did not exist in those with high narcissistic personality scores. Narcissism may act as a buffer against the effect of death anxiety on life satisfaction since narcissists can facilitate the active and passive accrual of their social networks [78]. Some studies have even shown a positive relationship between narcissism and well-being in the common social context [79].

The present study has some practical implications as well. It has been a long time since we humans encountered such public health challenges; death anxiety brought from the unexpected COVID-19 pandemic indeed damaged individuals’, and especially medical staff’s, mental health and psychological well-being [25]. However, the present findings showed that during a pandemic, feeling anxiety from life scarcity would enhance the thinking about the meaning of life, which may further help medical staff pull through and achieve their goals [80]. Additionally, understanding what personality traits may help buffer detrimental outcomes has unique implications for individuals. Whereas prior studies have focused on the dark side of narcissism, our study is a preliminary exploration that focuses on the bright side of narcissism. We provided an insight that research should not treat narcissism as a completely maladaptive personality, and that optimizing its adaptive aspects is beneficial for both personality and mental health.

We acknowledge that there are some limitations in interpreting the results of the present study. First, we employed snowball sampling for data collection. While useful when participants are hard to collect, the biggest setback of snowball sampling is that it is limited to a group of people with similar attributes, which has the probability of reducing the group representation. Moreover, we did not divide participants into doctors and nurses according to professional experience, so we cannot be certain if there would be a different pattern emerging between doctors and nurses. This was a cross-sectional study that could not reveal a temporal or causal relationship. Based on the high correlation between the two types of well-being, it is likely that subjective realities are intertwined. It was difficult to make this distinction in the present study because the data collection period only covered 3 weeks. A longitudinal panel study over a longer period of time would provide more insight. Another issue with the present study is that hedonic well-being was measured by the SWLS, which has been used in many studies as a measurement of subjective happiness [4]. However, people may gain life satisfaction through pleasure, engagement, and meaning [81]. Thus, it is possible that the life satisfaction measured in the present study overlaps with both hedonic and eudaimonic well-being, and the results could be a subject for further examination. We also acknowledge that although the results satisfied most of the hypotheses, the coefficients were weak. There may be other complex factors at work between death anxiety and well-being during the pandemic, and narcissism was a mild moderator that helped buffer adversity and maintain stable mental health.

## 5. Conclusions

There was a “psychological typhoon eye effect” during the COVID-19 pandemic, in which the medical staff in Wuhan, where the outbreak hit the hardest, showed less death anxiety than those in low-contagion regions. Our study also found a significant negative relationship between death anxiety and hedonic well-being, but not between death anxiety and eudaimonic well-being. In addition, the results revealed that a narcissistic personality moderates the relationships between death anxiety and the two types of well-being. For those who had higher narcissism, death anxiety had no negative effect on their well-being.

## Figures and Tables

**Table 1:** Descriptive Statistics and Correlation Analysis of the Present Study.

	*M*(*SD*)	Skewness	Kurtosis	1	2	3	4	5	6	7	8	9
1 Age	35.27(7.98)	----	----	1								
2 Sex	----	----	----	----	1							
3 Area	----	----	----	----	----	1						
4 Death anxiety	2.97(2.03)	0.63	−0.26	0.07	0.03	−0.21 **	1					
5 Hedonic well-being	4.11(1.48)	−0.04	−0.61	−0.09	−0.04	−0.12 **	−0.15 **	1				
6 Eudaimonic well-being	4.91(0.96)	−0.34	0.09	0.07	−0.04	−0.09	−0.06	0.51 **	1			
7 Presence of meaning	4.81(1.16)	−0.21	−0.13	0.04	−0.03	−0.08 *	−0.11 *	0.50 **	0.87 **	1		
8 Search for meaning	5.14(1.15)	−0.56	0.62	−0.02	−0.01	−0.04	−0.06	0.36 **	0.86 **	0.51 **	1	
9 Narcissistic personality	3.78(1.50)	−0.23	−0.59	0.02	−0.09 ^*^	0.06	0.27 **	−0.06	0.06	0.10 *	−0.03	1

Sex: “0” indicates male, “1” indicates female; Area: “0” indicates other area, “1” indicates Wuhan (the epidemic center). * *p* < 0.05, ** *p* < 0.01.

**Table 2:** Regression Model of Narcissism on Death Anxiety and Well-being (*n* = 705).

	Hedonic Well-Being			Eudaimonic Well-Being		
	Standardized Estimate	*t*	CI	Standardized Estimate	*t*	CI
Constant	4.93	20.35	[4.52 5.25]	5.30	43.74	[5.06 5.54]
Death Anxiety	−0.26	−3.41 **	[4.45 5.40]	−0.11	−1.92	[−0.29 0.09]
Narcissism	−0.14	−2.25 **	[−0.26 −0.02]	−0.03	−0.85	[−1.11 0.45]
Death Anxiety * Narcissism	0.06	2.84 **	[0.01 0.09]	0.03	1.99 *	[0.01 0.05]

* *p* < 0.05, ** *p* < 0.01. CI: confidence interval.

## Data Availability

Not applicable.
